# Characterization of germline development and identification of genes associated with germline specification in pineapple

**DOI:** 10.1038/s41438-021-00669-x

**Published:** 2021-11-01

**Authors:** Lihua Zhao, Liping Liu, Yanhui Liu, Xianying Dou, Hanyang Cai, Mohammad Aslam, Zhimin Hou, Xingyue Jin, Yi Li, Lulu Wang, Heming Zhao, Xiaomei Wang, Adrien Sicard, Yuan Qin

**Affiliations:** 1 College of Life Science, State Key Laboratory of Ecological Pest Control for Fujian and Taiwan Crops, Fujian Provincial Key Laboratory of Haixia Applied Plant Systems Biology, Center for Genomics and Biotechnology, Fujian Agriculture and Forestry University, Fuzhou, China; 2 Department of Plant Biology, Swedish University of Agricultural Sciences, Uppsala BioCenter and Linnean Centre for Plant Biology, Uppsala, Sweden; 3 State Key Laboratory for Conservation and Utilization of Subtropical Agro-Bioresources, Guangxi Key Lab of Sugarcane Biology, College of Agriculture, Guangxi University, Nanning, Guangxi China; 4 Horticulture Research Institute, Guangxi Academy of Agricultural Sciences, Nanning Investigation Station of South Subtropical Fruit Trees, Ministry of Agriculture, Nanning, China

**Keywords:** Flowering, Non-model organisms

## Abstract

Understanding germline specification in plants could be advantageous for agricultural applications. In recent decades, substantial efforts have been made to understand germline specification in several plant species, including Arabidopsis, rice, and maize. However, our knowledge of germline specification in many agronomically important plant species remains obscure. Here, we characterized the female germline specification and subsequent female gametophyte development in pineapple using callose staining, cytological, and whole-mount immunolocalization analyses. We also determined the male germline specification and gametophyte developmental timeline and observed male meiotic behavior using chromosome spreading assays. Furthermore, we identified 229 genes that are preferentially expressed at the megaspore mother cell (MMC) stage during ovule development and 478 genes that are preferentially expressed at the pollen mother cell (PMC) stage of anther development using comparative transcriptomic analysis. The biological functions, associated regulatory pathways and expression patterns of these genes were also analyzed. Our study provides a convenient cytological reference for exploring pineapple germline development and a molecular basis for the future functional analysis of germline specification in related plant species.

## Introduction

In sexually reproducing organisms, the establishment of the germline initiates the transition toward the haploid phase of the life cycle and ultimately leads to the production of gametes. Understanding how the germline initiates and develops can assist in the generation of useful breeding tools that could be used to produce male-sterile lines, promote heterosis, or develop apomictic plants. In most flowering plants, germline cells originate from somatic cells during the transition from the vegetative to reproductive phase^1,2^. The female germline develops within the ovule, whereas the male germline matures within the anther^3,4^. In angiosperms, both male and female germline development can be divided into two stages: microsporogenesis and microgametogenesis leading to the formation of male gametes and megasporogenesis and megagametogenesis giving rise to female gametes. During megasporogenesis, the nucellus of the ovule produces an archespore cell (AC) from a single hypodermal somatic cell, which further develops into a megaspore mother cell (MMC). Within the anther, microsporogenesis are initiated in multiple hypodermal somatic cells to produce several ACs, which all undergo periclinal and anticlinal cell divisions to generate pollen mother cells (PMCs). The initiation of these processes at particular positions within the sporophyte, referred to as germline specification, has been shown to be tightly regulated by diverse regulatory pathways in the plant model Arabidopsis thaliana (hereafter referred to as *Arabidopsis*).

The MADS-box homeotic gene *AGAMOUS* (*AG*) initiates germline specification by activating the SPOROCYTELESS/NOZZLE (SPL/NZZ) transcription factor in the ovule and anther primordia^5^. *SPL/NZZ*, in turn, activates the homeotic gene *WUSCHEL* (*WUS*), which regulates ovule patterning and initiates MMC formation^6^. *WUS* promotes MMC formation by indirectly activating the expression of the small WINDHOSE 1 and 2 (WIH1/2) peptides, which together with the tetraspanin-type protein TORNADO2 (TRN2) and the leucine rich-repeat protein TRN1 promote megasporogenesis^7^. The expression of *WUS* and *SPL/NZZ* is also promoted by the receptors of the plant hormone cytokinin receptors ARABIDOPSIS HISTIDINE KINASE4/CYTOKININ RESPONSE1 (AHK4/CRE1), AHK2 and AHK3. In addition, *SPL/NZZ* activates the expression of the auxin transporter gene *PIN-FORMED1* (*PIN1*), establishing a specific phytohormone gradient required for germline initiation^8,9^.

The small RNA-dependent silencing pathway plays an essential role in the specification of MMC^10^. *Arabidopsis* ARGONAUTE 9 (AGO9), a component of the RNA-directed DNA methylation (RdDM) pathway, acts autonomously in a non-cell manner in association with 24-nucleotide short interfering RNAs (siRNAs) to limit the formation of multiple MMCs and silence transposable elements (TEs)^11^. Mutations in other AGO genes, such as *AGO4*, *AGO6* and *AGO8*^12^, and other RdDM components, such as *RNA-DEPENDENT RNA POLYMERASE 6* (*RDR6*), *SUPPRESSOR OF GENE SILENCING3* (*SGS3*), *DICER-LIKE 3* (*DCL3*), *NUCLEAR RNA POLYMERASE D1A* (*NRPD1A*), and *NRPD1B*^11^, also cause the formation of multiple MMC-like cells. We recently showed that the spatial restriction of the biogenesis and mobility of a *TAS3*-derived trans-acting small interfering RNA (tasiRNA) targeting AUXIN RESPONSE FACTORs (ARF), tasiR-ARF, restricted MMC differentiation to a single cell^13,14^. Mutations of tasiR-ARF biogenesis components, including *TRANSCRIPTION EXPORT 1 (TEX1)*, *SUPPRESSOR OF GENE SILENCING 3 (SGS3)* and *AGO7*^14,15^, were observed in multiple MMC-like cells^13,14^.

Another factor regulating MMC specification is the cytochrome P450 monooxygenase KLUH (KLU), which guides the incorporation of H2A. Z by the chromatin remodeling complex SWR1 at the *WRKY28* locus to spatially restrict germline differentiation^15^. Cell cycle regulators such as the cyclin-dependent kinase inhibitors KIP-RELATED PROTEINs/INHIBITORS OF CYCLIN-DEPENDENT KINASES (KRPs/ICKs)^8,16^, RETINOBLASMA-RELATED PROTEIN 1 (RBR1), the RNA helicase MNEME (MEM) and the phytohormones auxin and cytokinin^17^ were also shown to contribute to the specification of germlines in *Arabidopsis*^15,18^. Mutants of these genes cause defects in germline cell fate specification, resulting in several or no MMC formations per ovule^15,19–21^.

Similarly, several key regulators of male germline specification have been identified. In *Arabidopsis*, the *TAPETUM DETERMINANT1* (*TPD1*) ligand and its leucine-rich repeat receptor-like kinases *(LRR-RLKs) EXCESS MALE SPOROCYTES1/EXTRA SPOROGENOUS CELLS* (*EMS1/EXS*) and *SOMATIC EMBRYO RECEPTOR KINASE 1/2* (*SERK1/2*) are essential for male germline cell fate determination and differentiation^22–27^. The rice *TDL1A-MULTIPLE SPORCYTE1 (MSP1)* ligand-receptor pair, homologs of *Arabidopsis TPD1-EMS1/EXS*, and the maize MALE STERILE CONVERTED ANTHER1 (MSCA1) glutaredoxin and small protein MULTIPLE ARCHESPORIAL CELLS1 (MAC1) also play essential roles in germline specification and differentiation^16,28–33^. The rice AGONAUTE protein MEIOSIS ARRESTED AT LEPTOTENE 1 (MEL1), RNA-recognition-motif protein MEIOSIS ARRESTED AT LEPTOTENE2 (MEL2), and maize protein AMEIOTIC1 *(*AM1) regulate premeiotic mitosis and meiosis initiation^34,35^. Mutants of several *RECEPTOR-LIKE KINASE* (*RLK*) genes, including *rice MSP1, Arabidopsis BAM1/2, Arabidopsis SERK1/2* and *Arabidopsis EMS1*, have excessive PMC-like cells and lack somatic layers, including the endothecium, middle layer, and tapetum^36^. Although several regulators of germline initiation have been studied in model plants such as *Arabidopsis*, little is known about the regulation of these processes in important crop species such as pineapple.

Pineapple (*Ananas comosus* (L.) Merr.) is a nonclimacteric tropical fruit that has outstanding nutritional and medicinal properties. As a member of the Bromeliaceae family, pineapple diverged from the lineage leading to grasses (Poaceae) ~100 million years ago. Due to the high self-incompatibility of pineapple, 15 to 16 years are typically required to breed and release a new cultivar^37^. Requests from farmers, vendors, and consumers to improve fruit quality and other agronomic traits are continuously increasing. Therefore, understanding the fundamental reproductive biological processes and developing advanced breeding techniques using new genetic, genomic, and biotechnological tools to breed new varieties have become very important^38^. Moreover, with excellent genetic resources and a fully sequenced genome^39,40^, pineapple has become an ideal system for studying the molecular mechanisms of germline development in exotic fruit crops.

In this study, correlations between pineapple floral organ sizes and germline developmental stages were assessed, chromosomal behavior during meiosis was observed, and genes preferentially expressed at the MMC and PMC stages were identified. The biological functions, associated regulatory pathways and expression patterns of the genes preferentially expressed in MMC-stage ovules and PMC-stage anthers were also analyzed. This study provides a cytological basis for germline development and demonstrates how these results constitute a powerful tool for elucidating the molecular mechanisms controlling pineapple germline specification when combined with comparative transcriptomics.

## Results

### Phenology of pineapple flower

**Fig. 1:**
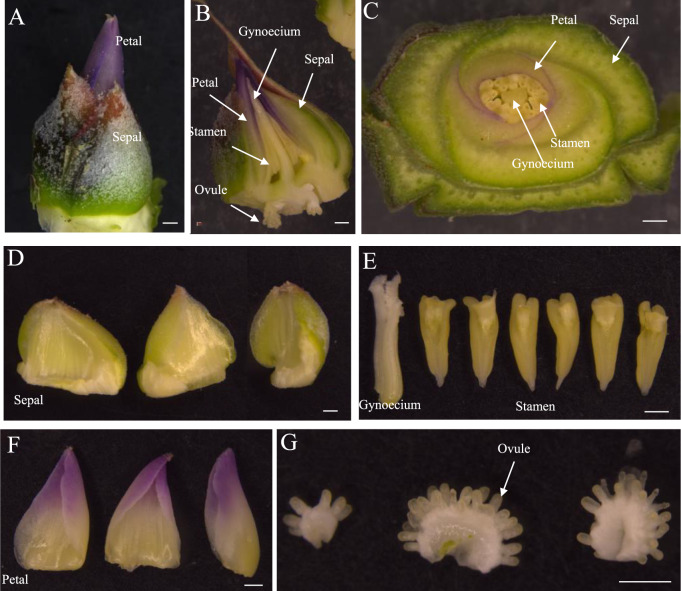
Phenology of pineapple flower. **A** A pineapple flower at the preflowering stage. **B** Longitudinal section of a flower. **C** Cross-section of a flower. **D** Three sepals. **E** Gynoecium and six anthers. **F** Three petals. **G** Ovules from three carpels within a flower. A–G Bars = 1 mm

The inflorescence of the pineapple MD2 variety usually consists of 50–80 individual flowers arranged in a compact spiral shape. Each flower contains three sepals, six stamens, one gynoecium and three purple petals (Fig. 1A–F). They have an inferior ovary with three carpels, and each carpel has ~20 ovules arranged in a fan-shaped placenta (Fig. 1G).

### Timeline of female germline development

To assess the correlations between germline development and floral organ development in pineapple, we systematically analyzed the developing flower morphology and characterized the corresponding female germline developmental stages. This analysis generated a reproductive timeline for the pineapple variety MD2 that allows the prediction of female germline developmental stages based on floral organ morphologies (Table 1 and Supplementary Fig. S1).

**Table 1:** Reproductive timeline: average floral bud size and gametogenesis in pineapple

Stage	Average width of floral bud (mm)	Average height of floral bud (mm)	Ovule development; female and male gametogenesis
1	3	2	Ovule primordium initiation; visible six anthers
2	4	2	Rapid growth of ovule primordium; differentiation of AC in the young anther
3	4	4	Differentiation of AC in ovule and the integument having similar height as the ovule primordium; PMC formation
4	5	4	MMC formation; PMC enters meiosis
5	6	5	Still in MMC stage; the inner integument covers the nucellus; pollen development at the tetrad stage
6	7	7	MMC prepares for meiosis; the out integument is longer than the inner integument; one-nucleate microspores
7	8	8	MMC enters meiosis stage; the out integument has reached the micropylar pole; one-nucleate microspores
8	8	8	FM formation; one-nucleate microspores
9	9	Petal just visible	Two-nucleate stage of female gametophyte; two-nucleate microspores
10	9	1 mm petal visible	Four-nucleate stage of female gametophyte; two-nucleate microspores
11	9	2.5 mm petal visible	Eight-nucleate stage of female gametophyte; three-nucleate microspores often visible
12	9	6 mm petal visible	Mature female gametophyte; mature pollen grain

*AC* archesporial cell, *FM* functional megaspore, *MMC* megaspore mother cell, *PMC* pollen mother cell

**Fig. 2:**
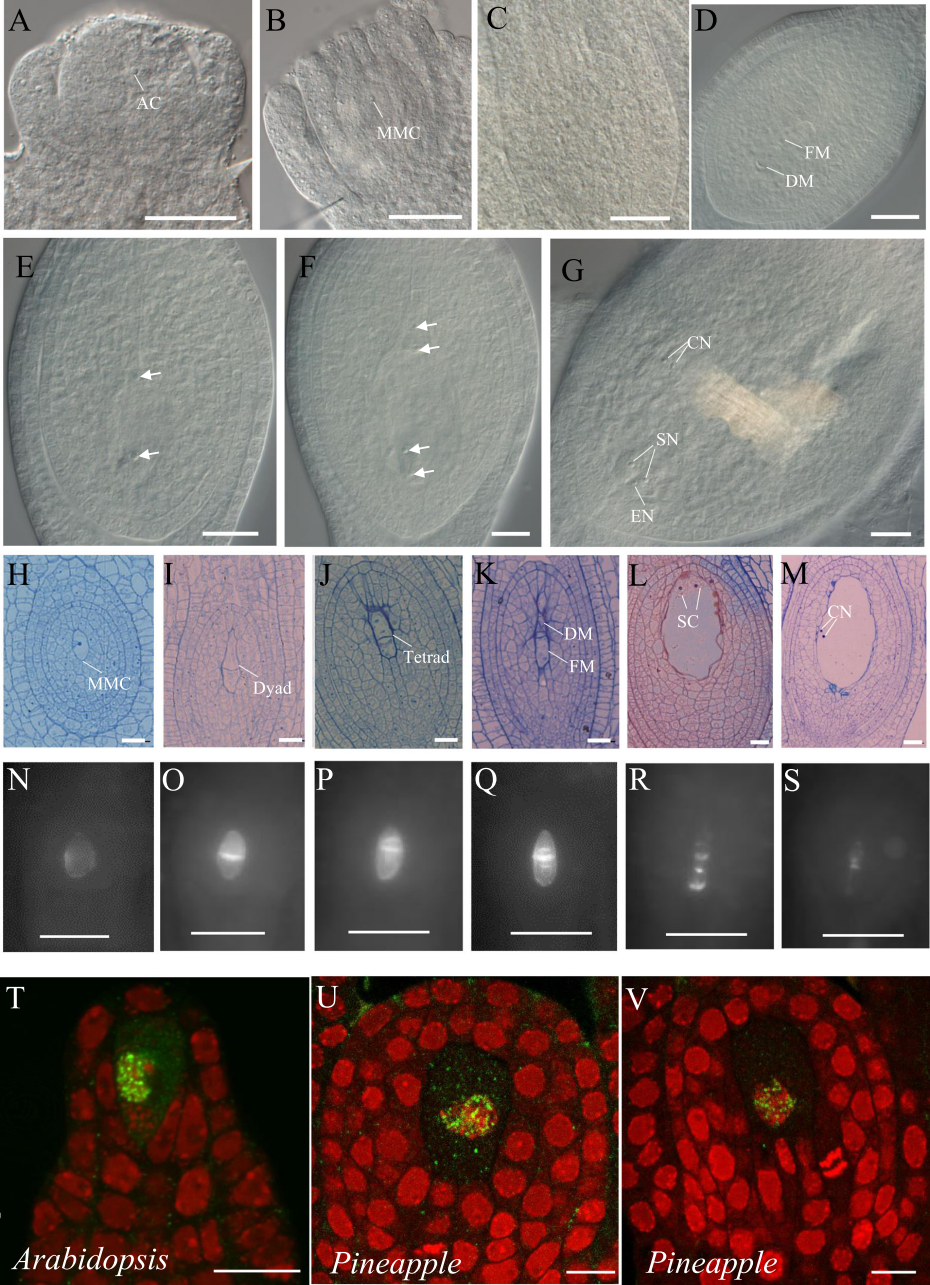
Female germline development in pineapple. **A**–**G** DIC observation of the ovule primordium at the AC (**A**), MMC (**B**), meiosis (**C**), and FM (**D**) stages; developing ovules at the two-nuclear (**E**) and four-nuclear stages of megagametogenesis (**F**); and a fully differentiated ovule containing a cellularized female gametophyte (**G**). **H**–**M** Semithin section of ovules at the MMC (**H**), dyad (**I**), tetrad (**J**), and FM (**K**) stages and fully differentiated ovules containing two SNs (**L**) and a CN (**M**). **N**–**S** Cell plate formation during megasporogenesis as revealed by callose staining. **T** Whole-mount immunolocalization analysis of an ovule in Arabidopsis using a DMC1 antibody. **U**–**V**. Whole-mount immunolocalization analysis of an ovule in pineapple using a DMC1 antibody. Green represents the DMC1 signal. AC archesporial cell, CN central cell nucleus, DM degenerated megaspore, EN egg cell nucleus, FM functional megaspore, MMC megaspore mother cell, SC synergid cell, SN synergid cell nucleus. **A**–**G**, Bars = 50 μm, **H**–**M**, Bars = 20 μm, **N**–**S**, Bars = 50 μm, **T**–**V**, Bars = 10 μm

The ovule primordium starts to initiate when the average floral buds size reaches ~3 mm in width and 2 mm in height. When the floral bud size reaches 4 mm in width and height, the AC differentiates at the distal pole of the ovule primordium, and the integument initiates (Fig. 2A). When it reaches 5 mm in width and 4 mm in height, the MMC is visible in the ovule primordium, and the integument elongates to a height similar to that of the nucellus (Fig. 2B, H). At 8 mm in width and height, the petals remain covered by sepals, and the MMC enters meiosis. At this stage, the MMC was not clearly observable by differential interference contrast (DIC) microscopy within the nucleus (Fig. 2C), which remained fully enveloped by integuments. A semithin section of the ovule revealed the formation of the dyad and tetrad (Fig. 2I, J). At a bud size of ~9 mm in width and 8 mm in height, one surviving cell of the tetrad differentiates into a functional megaspore (FM) (Fig. 2D, K). After that, the petals grow and gradually turn purple; the FM undergoes three mitotic nuclear divisions, followed by cellularization (Fig. 2E, F). The mature female gametophyte, which consists of two synergid cells, two central cells, and one egg cell at the micropylar pole, can be observed in flower buds, where the petals are ~2.5 mm longer than the calyx (Fig. 2G, L, M).

### Female meiosis progression and immunolocalization analysis of meiotic proteins in the ovule

In sexually reproducing organisms, meiosis is essential for the formation of gametes. We therefore used callose staining and whole-mount immunolocalization methods to characterize in detail the processes of meiosis during pineapple female germline development. These methods are routinely used to follow female meiosis progression in *Arabidopsis*^41–43^.

Callose staining is used to detect subsequent cytokinesis events during germline formation. Callose deposition at the cell plate after each cell division was detected by aniline blue staining. Before the start of meiosis, weak callose deposition was visible in the cell wall surrounding the MMC (Fig. 2N). Following the first cell division, a strong callose signal was observed in the newly formed cell plate (Fig. 2O). After the second cell division, two new callose bands resulting from the division of the daughter nuclei were observed above and below the first cell plate (Fig. 2P, Q). Later, callose bands degenerated gradually before disappearing at the end of meiosis (Fig. 2R, S). These observations suggest that cytokinesis progression during female meiosis in pineapple is similar to that in *Arabidopsis*^42^.

*DISRUPTION OF MEIOTIC CONTROL 1* (DMC1) is a conserved and essential protein involved in homologous chromosome pairing and recombination^44^. In *Arabidopsis*, DMC1 is specifically expressed in MMCs undergoing meiosis during ovule development. To determine whether DMC1 could also be used as an MMC marker in pineapple, we performed ovule whole-mount immunostaining using an antibody against the *Arabidopsis* DMC1 protein^45^. As in *Arabidopsis* (Fig. 2T), DMC1 signals were restricted to the MMC nucleus in pineapple ovules undergoing meiosis (Fig. 2U, V). This result suggested that DMC1 can be used as a marker of MMCs in pineapple and that our immunolocalization method can be used to examine protein localization during meiosis in pineapple ovules.

### Timeline of male germline development

**Fig. 3:**
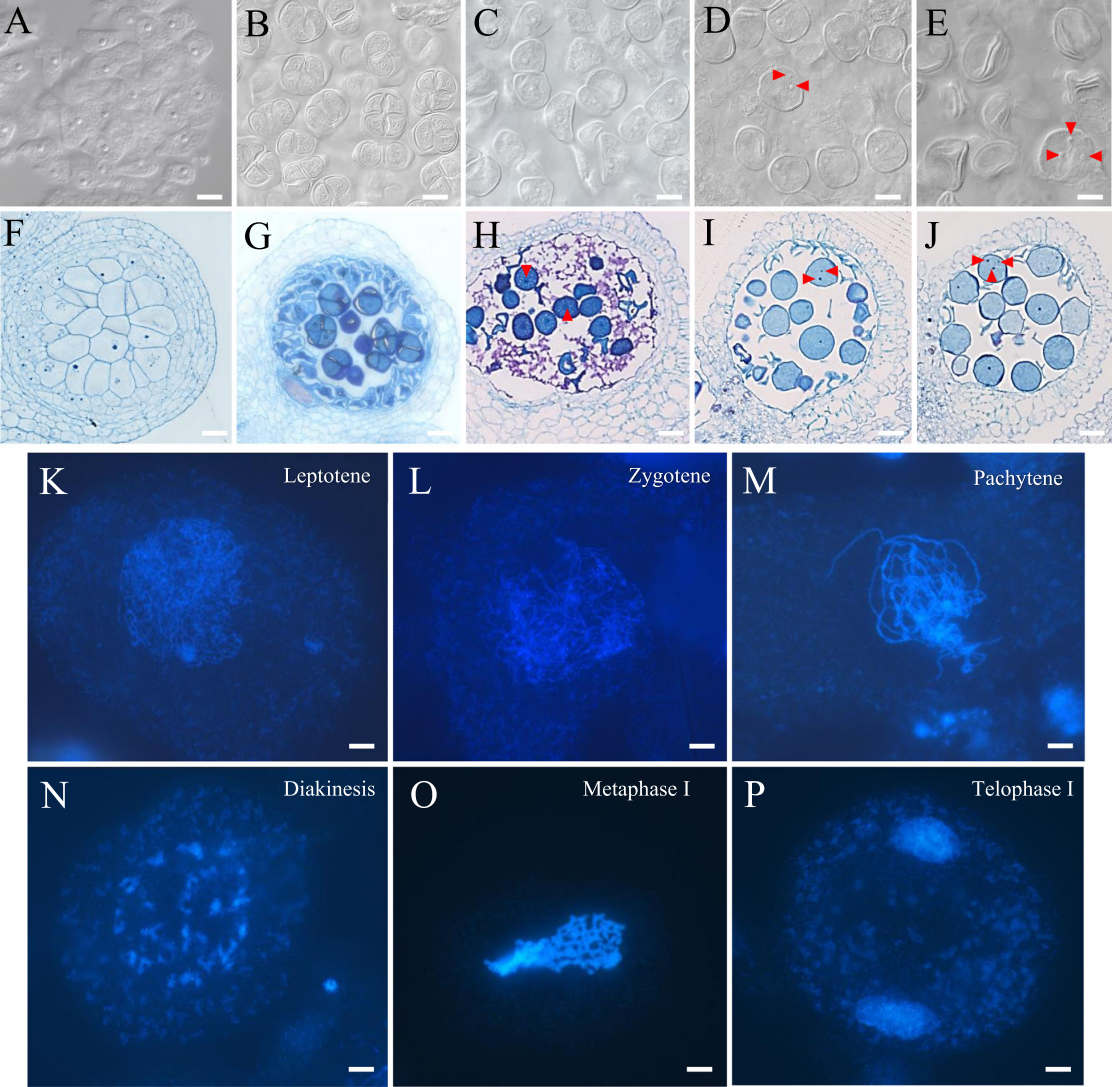
Male gametophyte development in pineapple. **A**–**J** DIC observations of semithin section of anthers at the pollen mother cell (**A**, **F**), tetrad (**B**, **G**), mononuclear pollen (**C**, **H**), bicellular pollen (**D**, **I**) and tricellular pollen (**E**, **J**) stages. **K**–**P**. Chromosome spreading of pollen mother cells undergoing meiosis at the leptatene (**K**), zygotene (**L**), pachytene (**M**), late diplotene and diakinesis (**N**), metaphase I (**O**) and telophase I (**P**) stages. Red arrowheads indicate the male gametophyte nucleus. **A**–**J**, Bars = 20 μm, **K**–**P**, Bars = 5 μm

The male germline development timeline was studied using DIC microscopic analysis of semithin sections (Table 1). We found that the PMC is formed when the bud size grows to 4 mm in width and height (Fig. 3A, F). When the floral bud reaches 5 mm in width and 4 mm in height, the PMC undergoes meiosis. Meiosis II completion and pollen tetrad formation occur when the floral bud size reaches approximately 6 mm in width and 5 mm in height (Fig. 3B, G). At the stage of pollen tetrad formation, the ovule reaches the MMC stage. When the floral bud size reaches ~7–9 mm in width, the mononuclear unicellular pollen is released from tetrads (Fig. 3C, H) and undergoes two rounds of mitosis to form bicellular pollen (Fig. 3D, I) and tricellular pollen (Fig. 3E, J), respectively.

We next studied chromosome behavior during male meiosis in pineapple using the chromosome spreading method. The typical morphology of chromosomes in different meiosis stages from prophase I to pollen tetrad was examined (Fig. 3K–P). In the leptotene stage, single and unpaired chromosomes are visible (Fig. 3K). In the zygotene stage, synapsis progresses, and chromosomes loop out, showing alternating synapsed and unsynapsed regions (Fig. 3L). In pachytene, the synapsis of homologous chromosomes is completed, as indicated by the thicker and shorter chromosomes (Fig. 3M). In late diplotene and diakinesis, 25 discrete and moderately condensed bivalents are visible (Fig. 3N). In metaphase I, the bivalents are more condensed and co-oriented on the spindle equator (Fig. 3O). In telophase I, two polar groups of chromosomes are separated (Fig. 3P), and eventually, tetrads of four microspores are formed. This meiosis atlas provides a basic outline of regular meiosis progression in pineapple and could serve as a useful framework to investigate the effects of different mutations on pineapple meiosis.

### Identification of genes preferentially expressed in MMC-stage ovules and PMC-stage anthers

We previously conducted RNA sequencing (RNA-seq) analysis of the pineapple ovule at 7 developmental stages (ovule 1–7) and of the pineapple stamen at 6 developmental stages (stamen 1–6)^46^. According to the flower bud width and length described above, Ovule_1 and Ovule_2 samples represent the MMC at stages 5 and 6 on the timeline, respectively, while Stamen_1 and Stamen_2 samples represent the PMC at stages 3 and 4, respectively on the timeline, Ovule_7 represents mature ovules at stage 12 on the timeline, and stamen_6 represents mature stamens at stage 11 on the timeline. To identify genes that are preferentially expressed in ovules of the MMC stage, we compared the transcriptomes of the Ovule_1 and Ovule_2 samples with the those of leaf, root, mature flower^47^, early-stage stamen (Stamen_1, Stamen_2), mature-stage stamen (Stamen_6) and mature stage ovule (Ovule_7) samples. These analyses identified 229 genes in Ovule_1 and Ovule_2 with fragments per kilobase of exon per million reads mapped (FPKM) values greater than 1 and two times higher than those of the other samples. These 229 genes were designated as MMC-stage ovule preferentially expressed genes (Supplementary Table S1). Similarly, the genes preferentially expressed in anthers of the PMC stage were identified by comparing the transcriptomes of Stamen_1 and Stamen_2 with those of the leaf, root, flower, ovule (Ovule_1, Ovule_2 and Ovule_7) and mature-stage stamen (Stamen_6) samples. This analysis identified 478 genes in Stamen_1 and Stamen_2 with FPKM values greater than 1 and two times higher than those of the other samples. These genes were designated as PMC-stage anther preferentially expressed genes (Supplementary Table S2).

**Fig. 4:**
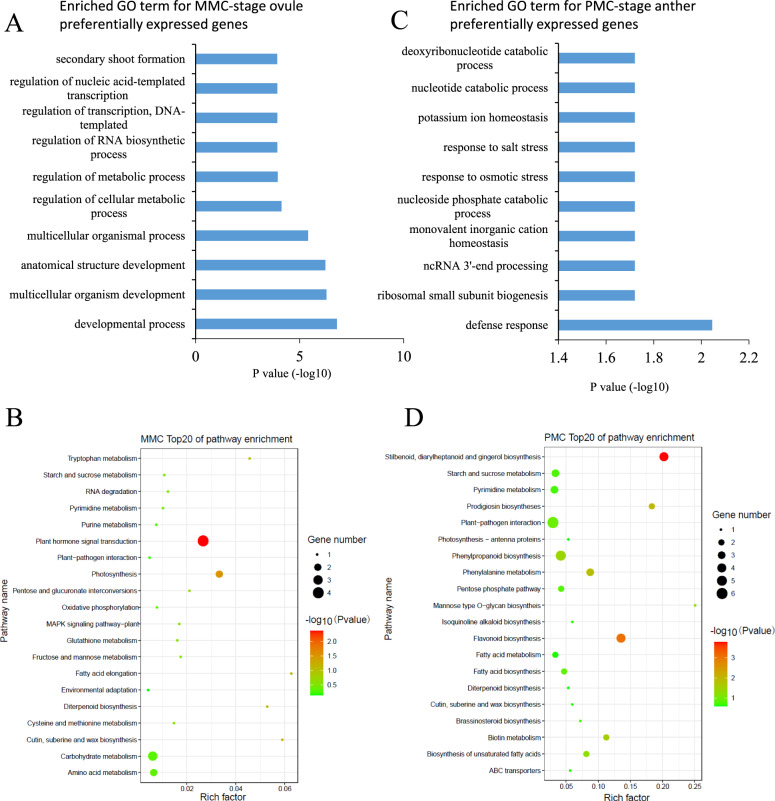
Enrichment analysis of genes preferentially expressed in MMC-stage ovules and PMC-stage anthers. **A** GO terms enriched in MMC-stage ovule preferentially expressed genes. **B** KEGG pathway enrichment in MMC-stage ovule preferentially expressed genes. **C** GO terms enriched in PMC-stage anther preferentially expressed genes. **D** KEGG pathway enrichment in PMC-stage anther preferentially expressed genes

After identifying the MMC- and PMC-stage preferentially expressed genes, we functionally classified them based on their biological or biochemical function using the gene ontology (GO) annotation of *Ananas comosus*. We found that GO terms related to development, metabolism, transcription, RNA biosynthesis, and secondary shoot formation were significantly overrepresented within the genes preferentially expressed in MMC-stage ovules (Fig. 4A). These findings suggested that ovule primordia are very active in transcription and metabolism during MMC specification. Furthermore, the key regulatory pathways in the early stage of pineapple ovule development were also subjected to Kyoto Encyclopedia of Genes and Genomes (KEGG) pathway analysis. Genes related to the plant hormone signal transduction, photosynthesis, MAPK signaling, cutin, suberin, and wax biosynthesis pathways were highly enriched in MMC-stage ovules (Fig. 4B). These results further indicated that plant hormones and signal transduction might play important roles in ovule primordia during MMC specification.

Moreover, the GO terms related to response to stress and ion homeostasis were enriched in genes preferentially expressed in PMC-stage anthers (Fig. 4C). KEGG analysis indicated that genes involved in the stilbenoid, diarylheptanoid and gingerol biosynthesis pathway; flavonoid biosynthesis pathway; prodigiosin biosynthesis pathway; and phenylalanine metabolism pathway were overrepresented in PMC-stage anther preferentially expressed genes (Fig. 4D). These results suggest that most of the biological functions and related pathways differ between MMC-stage ovules and PMC-stage anthers.

To increase the accuracy of the identified MMC- and PMC-stage preferentially expressed genes, we further screened the 229 identified MMC-preferred genes among the genes expressed in developing ovules (Ovule_3, Ovule_4, Ovule_5, and Ovule_6)^46^ and selected only those that had FPKM values that were at least two times higher than those in developing ovules. Using the same method, we compared 478 PMC-preferred genes with the genes expressed in developing anthers (Stamen_3 and Stamen_4)^46^. These analyses resulted in 21 and 167 genes specifically expressed in MMC-stage ovules and PMC-stage anthers, respectively (Supplementary Tables S3 and S4). Both the GO and KEGG analyses (Supplementary Tables S3 and S4) showed results similar to those obtained by the analyses above, suggesting that genes specifically expressed at the MMC and PMC stages are involved in the same biological processes.

### Expression patterns of the homologs of female germline specification genes in pineapple

**Fig. 5:**
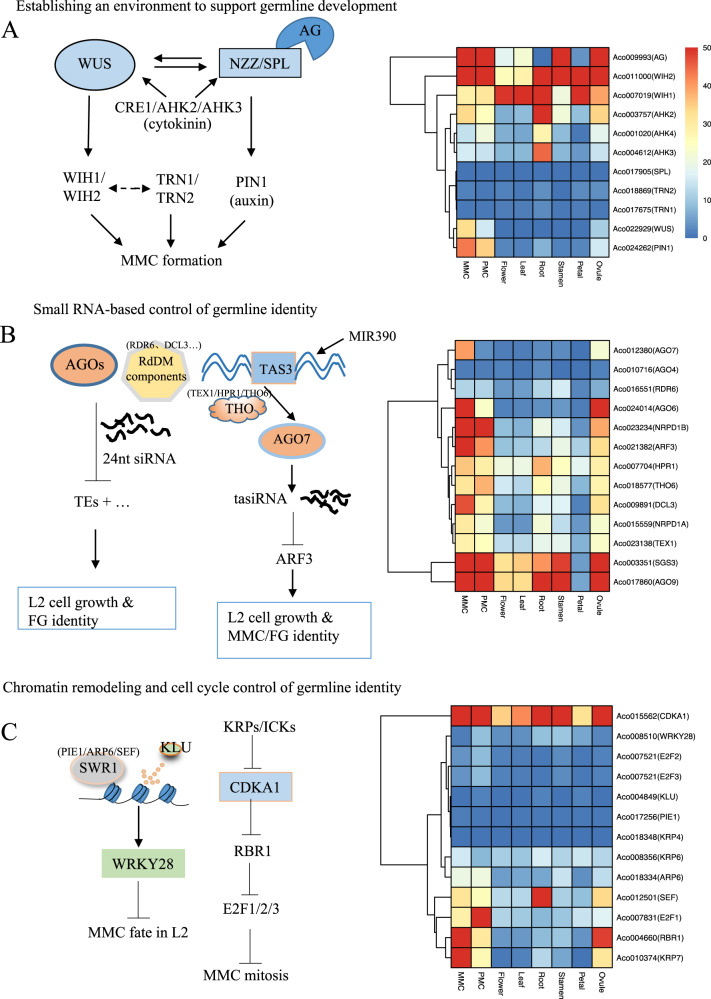
Expression patterns of the homologs of female germline differentiation genes in pineapple. **A** Expression pattern of members of the AG-NZZ/SPL signaling pathway in pineapple MMC-stage ovules. AG, AGAMOUS; WH1/2, WINDHOUSE 1 and 2; AHK2/3/4, ARABIDOPSIS HISTIDINE KINASES 2/3/4; SPL, SOPROCYTELESS/NOZZLE; TRN1/2, TORNADO2; WUS, WUSCHEL; PIN1, PIN-FORMED1. **B** Expression patterns of genes involved in the small RNA-dependent silencing pathway in pineapple MMC-stage ovules. AGO4/6/7/9, ARGOAUTE4/6/7/9; RDR6, RNA-DEPENDENT RNA POLYMERASE6; NRPD1A/B, NUCLEAR RNA POLYMERASE D1A/B; ARF3, ADP-RIBOSYLATION FACTOR 3; HPR1, THO6, TEX1, components of the THO/TREX complex; DCL3, DICER-LIKE 3; SGS3, SUPPRESSOR OF GENE SILENCING 3. **C** Expression patterns of genes in the SWR1-KLU- and KRP/ICK-mediated cell cycle regulation pathways in pineapple. CDKA1, CELL DIVISION CONTROL 2; WRKY28, WRKY DNA-BINDING PROTEIN 28; E2F1/2/3, E2F TRANSCRIPTION FACTORS 1/2/3; KLU, CYTOCHROME P450; PIE1, PHOTOPERIOD-INDEPENDENT EARLY FLOWERING 1; KRP4/6/7, KIP-RELATED PROTEINS 4/6/7; ARP6, ACTIN-RELATED PROTEIN 6; SEF, SERRATED LEAVES AND EARLY FLOWERING; RBR1, RETINOBLASTOMA-RELATED 1

To understand the molecular mechanisms controlling MMC specification in pineapple, we identified homologs of the genes involved in germline specification in *Arabidopsis thaliana* and examined their expression profiles using available transcriptomic data^46,48^. Within the *AG-NZZ/SPL* gene regulatory network (GRN), we found that *AcAG*, *AcWUS, AcPIN1* and *AcWIH2* were highly expressed in MMC-stage ovules and PMC-stage anthers. However, *AcSPL*, *AcTRN1* and *AcTRN2* were expressed at low levels in all of the assessed tissues (Fig. 5A). These results suggest that the *AG-NZZ/SPL* pathway is mostly conserved in pineapple and may promote its germline specification. The low expression of some components at the MMC and PMC stages may nevertheless suggest that different genes contribute to this pathway.

We next analyzed the expression of genes involved in the RdDM pathway, which plays an important role in germline specification. The majority of genes in this GRN, including *AcAGO7*, *AcAGO6*, *AcAGO9*, *Nuclear RNA polymerase D1B (AcNRPD1B)*, *ADP-ribosylation Factor 3 (AcARF3)*, *Endoribonuclease Dicer homolog 3 (AcDCL3)*, and *SUPPRESSOR OF GENE SILENCING 3 (AcSGS3)*, were shown to be highly expressed in MMC-stage ovules, indicating that small RNA-dependent silencing may also play a key role in MMC specification in pineapple (Fig. 5B).

The pineapple homologs of cell cycle regulators such as *AcCDKA1*, *AcRBR1* and *AcKRP7* were highly expressed in MMC-stage ovules (Fig. 5C). Members of the SWR1 complex, including *Actin-related protein 6* (*ARP6*) and *SERRATED LEAVES AND EARLY FLOWERING* (*SEF*), and the E2F family genes *E2F transcription factor 1* (*E2F1*)*, E2F transcription factor 2 (E2F2*) and *E2F transcription factor 3* (*E2F3*) displayed increased expression in PMC-stage anthers compared to other tissues (Fig. 5C). The conservation of the gene expression patterns suggests that the molecular pathways controlling germline specification are conserved in pineapple and highlights several candidate genes for further functional studies.

### Expression patterns of the homologs of male germline cell specification and meiotic genes in pineapple

**Fig. 6:**
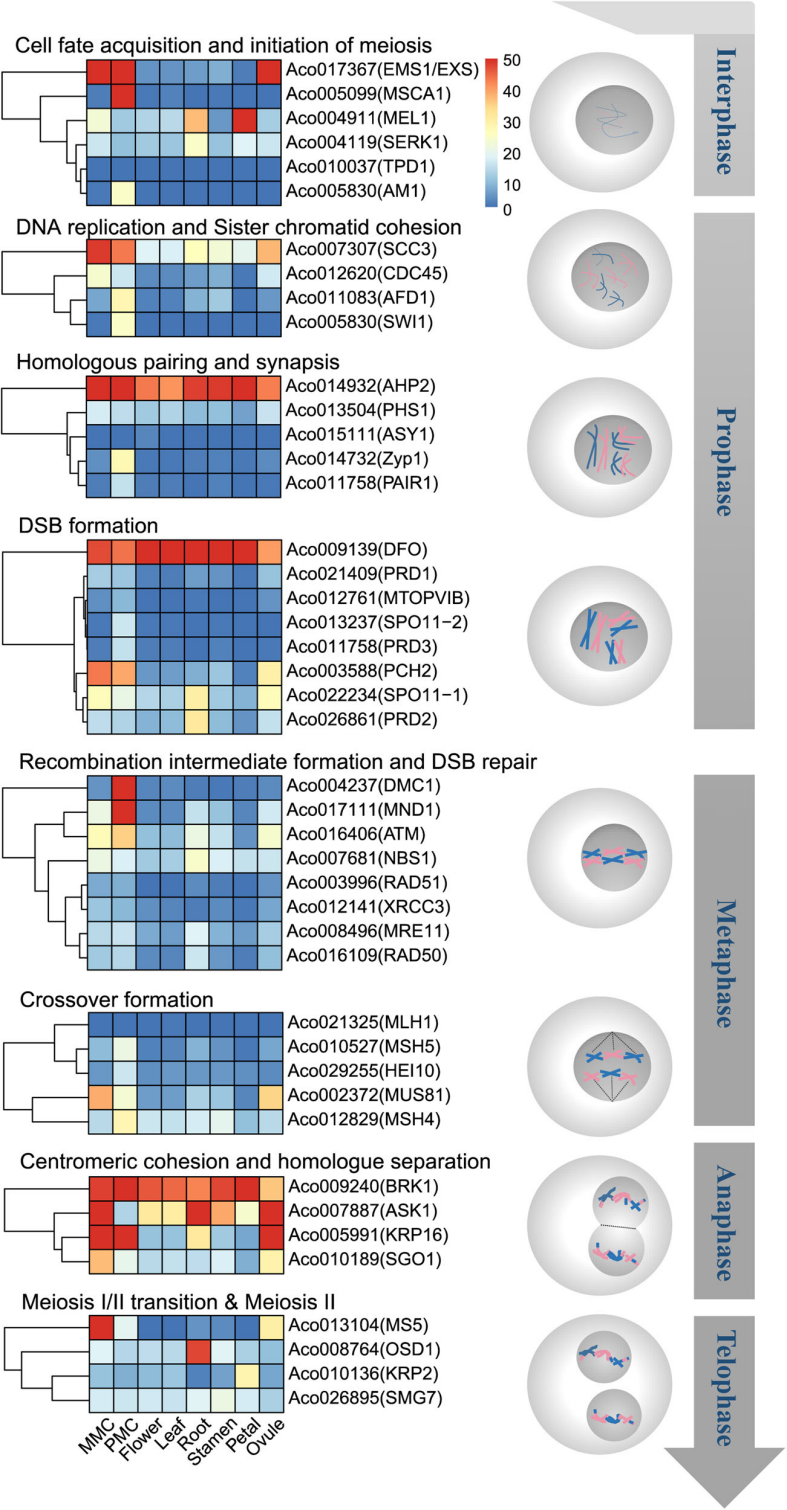
Expression patterns of genes related to cell fate acquisition and mitotic proliferation in pineapple. Heatmap of known meiotic genes during pineapple development. The genes are grouped according to their cellular function during meiosis

A useful tool for improving plant varieties is the manipulation of homologous recombination and genome haploidization during meiosis^49^. Meiotic recombination provides genetic diversity in populations and ensures accurate homologous chromosome segregation for genome integrity^50,51^. During meiosis, recombination processes ranging from DNA double-strand break (DSB) production to crossover formation are tightly linked to higher-order chromosome structural features, including chromatid cohesion, axial element formation, homolog pairing, and synapsis^50^. Therefore, we investigated whether the GRNs involved in male germline specification and meiosis are also conserved in pineapple by analyzing their expression profiles during pineapple male germline development (Fig. 6). Because meiosis is particularly important during microsporogenesis, we expected these genes to be highly expressed in PMC-stage anthers.

Genes involved in male germline acquisition and meiotic initiation, *AcEMS1*, *AcMSCA1* and *AcAM1*, were highly expressed in PMC-stage anthers. The homologs of the genes involved in DNA replication and sister chromatid cohesion, such as *AcSCC3*, *AcAFD1* and *AcSWI1*, and homologous pairing and synapsis, such as *AcZYP1* and *AcPAIR1*, were also highly expressed in PMC-stage anthers (Fig. 6). The homologs of many meiotic recombination genes in pineapple, such as *AcPCH2*, *AcDMC1*, *AcMND1*, *AcMSH5*, *AcHEI10* and *AcMSH4*, were highly expressed in PMC-stage anthers (Fig. 6). The high expression levels of these genes in PMC-stage anthers indicates that they may also play important roles in controlling meiotic recombination during pineapple anther development. Notably, most of the homologs of meiotic genes are also highly expressed in MMC-stage ovules, indicating the conservation of the molecular basis of meiosis between male and female germlines. Most of the pineapple homologs of genes functioning in late stages of meiosis, including *AcBRK1*, *AcASK1*, *AcOSD1*, *AcKRP2*, and *OcSMG7*, did not show preferential expression in MMC-stage ovules and PMC-stage anthers, suggesting that the molecular control of late-stage meiosis might be similar to that of mitosis.

### Members of specific transcription factor families showed enriched expression in MMC-stage ovules and PMC-stage anthers

**Fig. 7:**
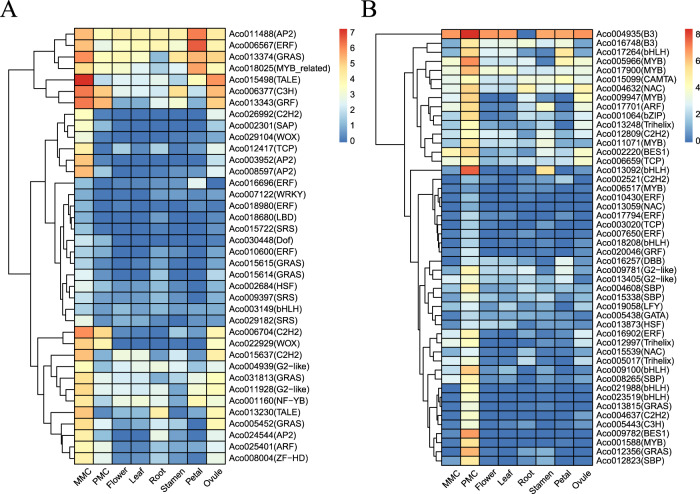
Members of specific transcription factor families that were enriched in MMC-stage ovules and PMC-stage anthers. **A**, **B** Heatmap of relative expression levels of the transcription factors preferentially expressed in MMC-stage ovules (**A**) and PMC-stage anthers (**B**)

Transcription factors (TFs) play key roles in diverse aspects of plant development, including germline development^17^. To identify the GRN controlling germline specification, we generated a heatmap of the TFs preferentially expressed during early ovule and anther development. We showed that 38 and 48 TFs were highly expressed in MMC-stage ovules and PMC-stage anthers, respectively (Fig. 7A). These TFs belong to multiple transcription factor families, including the *AP2* family, ethylene-responsive transcription factor *(ERF)* family, *GRAS* family, and *C2H2* family (Supplementary Tables S5 and S6). The high expression levels of these genes suggest that they play important roles in germline specification. In agreement with this idea, many of their homologs are required for gametophyte development in other plant species. For example, the *AP2* family member *AINTEGUMENTA* (*ANT*) plays a critical role in regulating ovule and female gametophyte development in *Arabidopsis*^52^. The *GRAS* family members *SlGRAS7* and *SlGRAS40* are involved in the flowering time and reproductive development of tomato^53,54^. The orthologs of the pineapple genes *Aco007650*, *Aco010430* and *Aco16902* belonging to the *ERF* family are essential for tapetum and microspore development^55^. In addition, the *bHLH*^56,57^, *MYB*^58^, *NAC*^59^, and *BES1*^60,61^ TFs regulate early anther development in both *Arabidopsis* and rice.

### Confirmation of gene expression patterns by qRT–PCR and in situ hybridization

**Fig. 8:**
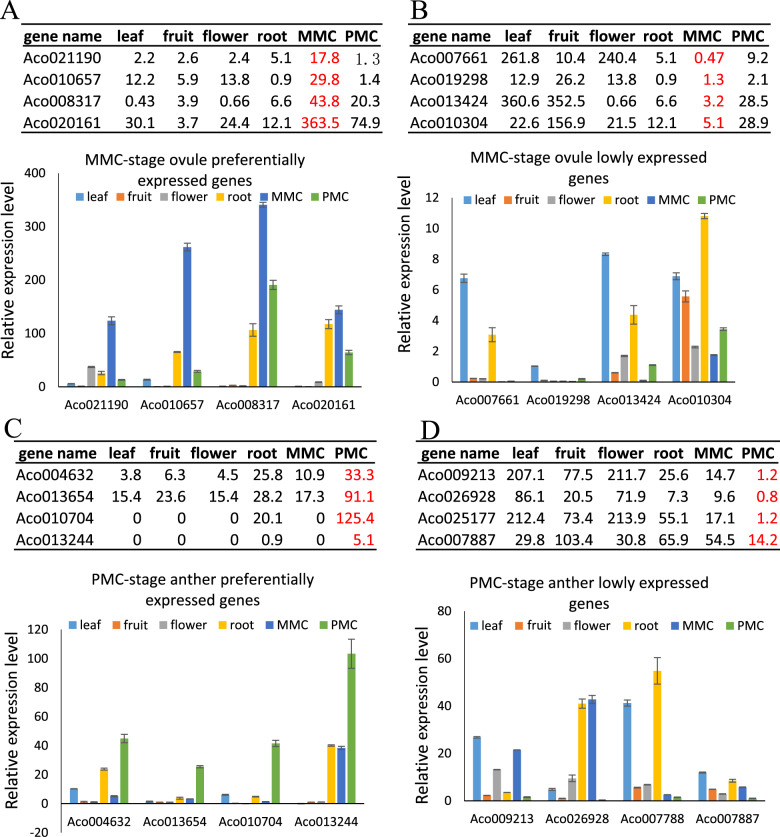
Quantitative RT–PCR analysis of 16 randomly selected genes. **A**, **B** Expression levels of differentially expressed genes (DEGs) in MMC-stage ovules as determined by RNA-seq and real-time qRT–PCR. **A** Upregulated genes in MMC-stage ovules. **B** Downregulated genes in MMC-stage ovules. **C**–**D** Expression levels of DEGs in PMC-stage anthers as determined by RNA-seq and real-time qRT–PCR. **C** Upregulated genes in PMC-stage anthers. **D** Downregulated genes in PMC-stage anthers. The table shows the RPKM values from RNA-seq, and the histograms display the relative expression levels of the genes in different pineapple tissues as determined by qRT–PCR

To validate the transcriptomic analysis, we next assessed the expression patterns of genes preferentially expressed in MMC-stage ovules and PMC-stage anthers in different tissues, including leaf, fruit, mature flower, root, MMC-stage ovules, and PMC-stage anthers, using semi-quantitative expression analysis (real-time reverse transcription PCR). For this analysis, we randomly selected four genes preferentially expressed in MMC-stage ovules, four genes expressed at low levels in MMC-stage ovules, four genes preferentially expressed in PMC-stage anthers, and four genes expressed at low levels in PMC-stage anthers (Fig. 8A–D). Consistent with the RNA-seq study, all eight genes preferentially expressed or expressed at low levels in MMC-stage ovules had high or low expression levels in MMC-stage ovules (Fig. 8A, B) (Supplementary Table S7). Similarly, the expression levels of the genes preferentially expressed or expressed at low levels in PMC-stage anthers as determined by real-time RT–PCR were highly consistent with the RNA-seq results (Fig. 8C, D). In addition, we confirmed the high expression levels in MMC-stage ovules for four homologs of genes known to be involved in germline specification (Supplementary Fig. S3).

**Fig. 9:**
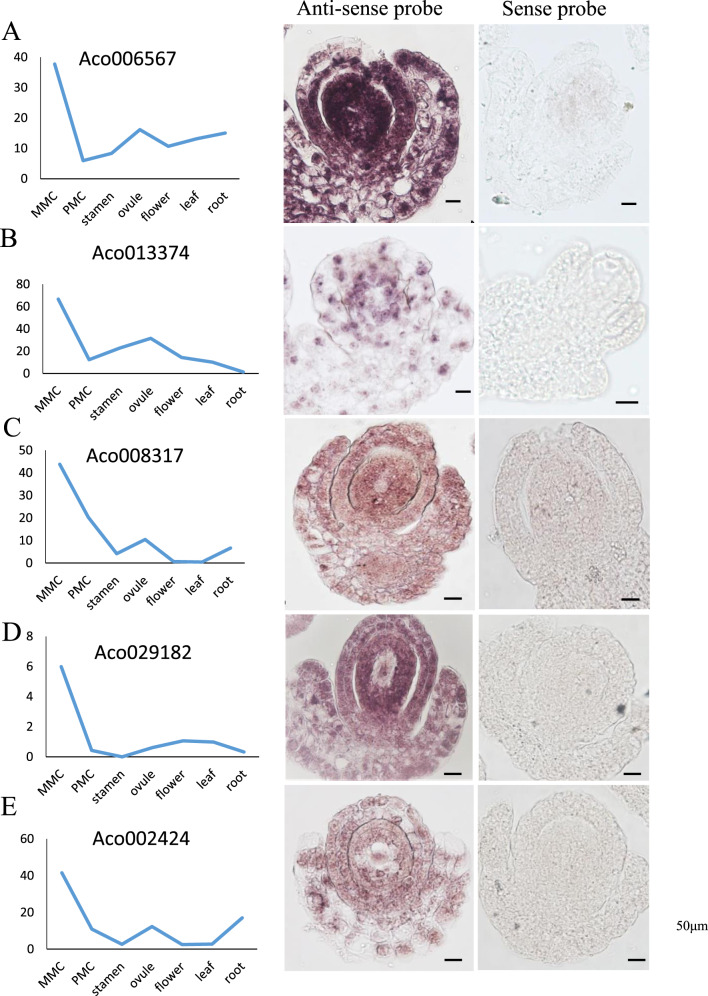
In situ hybridization analysis of candidate genes in MMC-stage ovules. Expression pattern of Aco006567 (**A**), Aco013374 (**B**), Aco008317 (**C**), Aco029182 (**D**), Aco002424 (**E**). Expression levels of candidate genes on RNA-seq were shown in the left panel. Representative images of sense and antisense RNA probe hybridization were shown in the right panels, Bars = 50 μm

**Fig. 10:**
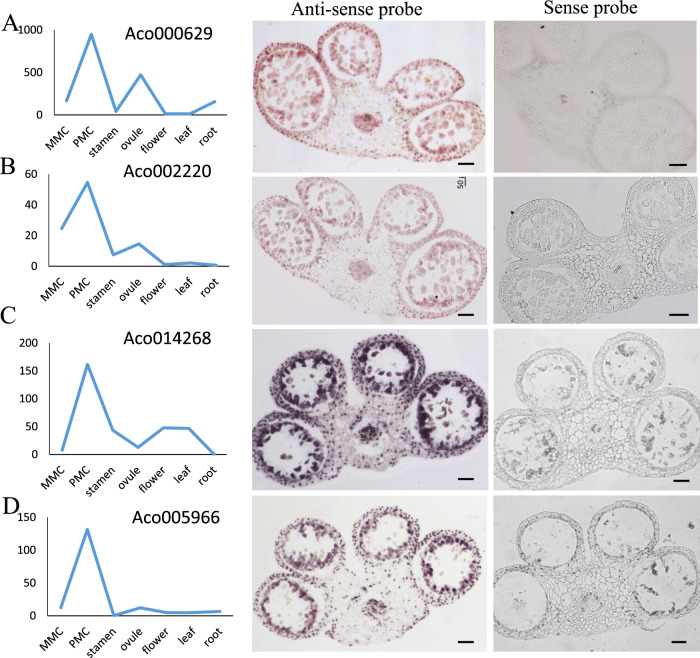
In situ hybridization analysis of candidate genes in PMC-stage anthers. Expression pattern of Aco000629 (**A**), Aco002220 (**B**), Aco014268 (**C**), Aco005966 (**D**). Expression levels of candidate genes on RNA-seq were shown in the left panel. Representative images of sense and antisense RNA probe hybridization were shown in the right panels, Bars = 50 μm

The expression patterns of nine MMC- or PMC-stage preferentially expressed genes were further validated by in situ hybridization. The MMC-stage preferentially expressed genes included an ethylene-responsive transcription factor *(Aco006567)*, a *GRAS* family transcription factor *(Aco013374)*, an *ARM* repeat superfamily protein *(Aco008317)*, *SHI*-*RELATED SEQUENCE 5 (Aco029182)*, and a protein kinase superfamily protein *(Aco002424)*. All five genes exhibited enriched expression in the nucellus of MMC-stage pineapple ovules, with *Aco006567* showing the strongest expression in the ovule (Fig. 9A–E). We also performed in situ hybridization analysis of four PMC-stage preferentially expressed genes encoding an annexin-like protein *(Aco000629)*, a *BZR1* family protein *(Aco002220)*, an ethylene-responsive transcription factor *(Aco014268)*, and a *MYB* domain protein 84 *(Aco005966)*. *Aco000629* and *Aco002220* were mainly expressed in the four lobes of nonreproductive tissue layers and microsporocytes (Fig. 10A, B). The expression of the other two genes, *Aco014268* and *Aco005966*, was mainly concentrated in the microsporocytes (Fig. 10C, D). No hybridization signal was detected with the control sense probes (Figs. 9, 10). These results further validated the reliability of our transcriptomic approach for identifying MMC- and PMC-stage preferentially expressed genes.

## Discussion

Although pineapple is a high-value crop of major economic importance for many countries, developing new high-performing cultivars remains time-consuming and labor-intensive because of its high degree of self-incompatibility. Compared to *Arabidopsis* and rice, knowledge about pineapple germline specification remains limited^62^. We recently performed a transcriptomic analysis of pineapple floral organs and explained the morphological characteristics of different floral organs^46^. The current study provides a detailed timeline of germline developmental stages during flower development and cytological methodologies to further elucidate the molecular mechanisms regulating pineapple sporogenesis. We further demonstrate how combining this knowledge with transcriptomic analyses can be used to improve our understanding of pineapple germline specification.

### Identification of genes specifically expressed during pineapple germline specification by transcriptome analysis

We performed a comparative transcriptomic analysis of different pineapple tissues. Based on our developmental analysis, the Ovule_1 and Ovule_2 samples correspond to the MMC at stages 5 and 6, respectively, and the Stamen_1 and Stamen_2 samples represent the PMC at stages 3 and 4, respectively. By comparing the transcriptomes of these stages with those of other developmental stages, we identified 229 and 478 genes preferentially expressed in MMC-stage ovules and PMC-stage pollen, respectively. Functional classification of these genes by KEGG and GO enrichment analyses revealed that the genes preferentially expressed in MMC-stage ovules belong to the “development process” and “multicellular organism development process” GO terms (Fig. 4). However, in rice, the enriched pathways for upregulated genes at the MMC stage were involved in “plant hormone signal transduction pathways” and “plant-pathogen interaction pathways”^63^. These differences may suggest that different regulatory pathways are involved in the specification of MMCs in different plant species. Nevertheless, functional classifications of the genes with enriched expression in MMC-stage ovules of rice and pineapple were correlated with the “hormone signal transduction” and “biosynthesis of secondary metabolites” pathways. These findings suggest that phytohormones and numerous biosynthetic activities are critical for MMC specification and conserved among plant species. In line with this argument, the inactivation of *TRAF-LIKE* genes, which alters the biosynthesis and transport of lipids, ultimately leads to aberrant megaspore mother cell specification in *Arabidopsis*^64^. Furthermore, we identified homologous genes involved in pathways known to mediate megaspore mother cell specification in other species, including the *AG-NZZ/SPL*, small RNA-dependent silencing and *KRP/ICK-*mediated cell cycle regulation pathways (Fig. 5). Most of these homologs showed similar expression profiles across different species, suggesting that the function of these pathways in MMC specification is conserved across the plant kingdom. For example, in accordance with previous studies in rice, we found that the auxin transporter protein PIN1, located downstream of the AG-NZZ/SPL signaling pathway, is especially highly expressed in pineapple MMC-stage ovules^63^. In *Arabidopsis*, a mutant of *CDKA1*, was reported to impair endosperm development, resulting in seed abortion^65^. The *rbr1* mutant showed a supernumerary nucleus in female gametophytes, contributing to plant fertility defects^66^. Interestingly, we also found that *AcCDKA1* and *AcRBR1* and *AcKRP7* were preferentially highly expressed in pineapple MMC-stage ovules in pineapple. Further investigation will nevertheless be required to characterize the roles of these genes during pineapple development. The detailed functions of these genes in pineapple MMC development need to be further investigated and may be applied to breed new pineapple varieties.

### Expression patterns of meiotic genes and transcription factors highlight their potential roles in pineapple germline specification

Meiosis, which produces haploid cells through specialized cell division, marks the transition from sporophyte to gametophyte generation in the life cycle of plants^67^. The establishment and maintenance of the fate of meiotic cells require intricate regulation of gene expression. Thus, distinct patterns of gene expression are a hallmark of sexual reproduction^68^. In line with their roles in germline cell fate decisions and meiosis initiation, homologs of genes associated with male germ cell specialization, such as *AcEMS1/EXS (Aco017367), AcMEL1 (Aco004911)*, and *AcSERK1 (Aco004119)*, were also highly expressed in MMC-stage ovules in pineapple (Fig. 6). During pineapple meiosis, genes responsible for meiotic recombination, such as *DMC1* and *MND1*, are preferentially expressed in PMC-stage anthers. In contrast, genes involved in the late stage of meiosis were weakly expressed in the MMC-stage ovules and PMC-stage anthers (Fig. 6). However, *DMC1* in wheat was previously reported to maintain normal chromosome synapsis and crossover during later stages of meiosis^69^, and the analysis of *OsDMC1-RNAi* lines demonstrated that *OsDMC1* was essential for rice homologous pairing^70^. These findings suggest that gene expression during meiosis is intricately regulated spatially and temporally, which might be critical for ensuring that the chronology of meiosis meiotic events proceeds properly in the subsequent stages. The similarities and differences in the functions of these genes during MMC and PMC specification require further investigation in the future.

Transcription factors play key roles in initiating developmental programs, including germline specification. Here, we identified a total of 38 transcription factors that were highly expressed in MMC-stage ovules and 48 transcription factors that were highly expressed in PMC-stage pollen (Fig. 7). The transcription factors with higher expression in MMC-stage ovules included five *GRAS* family members, four *AP2* family members, and four *ERF* family members (Fig. 7A). In rice, members of the *MYB, bZIP, bHLH, WRKY*, and *MADS* TF families showed enriched expression in MMC-stage ovules^63^. Consistently, we also found that some TFs belonging to the *MYB, AP2*, and *bHLH* families showed high expression in pineapple MMC-stage ovules. Interestingly, the role of *GRAS* family members in ovule development has not been reported, suggesting that the GRNs regulating germline specification have functionally diversified among plant species. Further characterization of the five GRAS transcription factors found to be preferentially expressed in pineapple MMC-stage ovules will shed light on their function during MMC specification. Six *MYB* family members, six *bHLH* family members, and four *ERF* family members were among the transcription factors with increased expression in PMC-stage anthers (Fig. 7B). In a previous study, network component analysis (NCA) was used to identify TF activities, revealing that several TFs from the families mentioned above, including *WRKY34, ERF6, and MYB65*, play key roles in pollen development^71^. In *Arabidopsis*, the second largest family of dimerizing TFs, bZIPs, interacts with a GRN network involved in pollen development^72^, suggesting the conserved and complicated roles of several TFs in PMC development in plants.

### The ethylene signaling pathway might be involved in pineapple germline development

Phytohormones are widely known to be associated with plant growth and development. Our transcriptomic analysis also indicated that phytohormone signal transduction pathways were prominently enriched in the MMC-stage ovules (Fig. 4B). Ethylene is a rather unusual plant hormone that acts in a gaseous form to regulate diverse developmental processes, including those involved in plant reproduction. Ethylene precursor 1-aminocyclopropane-1-carboxylic acid (ACC) is associated with pollen tube attraction in ovular sporophytic tissue^73^. Moreover, egg cell fertilization predominantly activates ethylene signaling^74^, and ethylene is required to initiate ovary development and subsequent ovule differentiation^75^. Recently, the potential function of *ERFs* in grape ovule development was reported^76^. In addition*, RhERF113* was reported to be induced by ethylene in rose stamens and pistils^77^. Evolutionary analysis suggests that *ERFs* have highly conserved roles in different species^76,78^. In agreement with the idea that the ethylene signaling pathway is involved in pineapple germline development, we identified several ERF transcription factor family members that were preferentially expressed in the MMC-stage ovules or PMC-stage stamen. Furthermore, our in situ hybridization assays revealed that the *ERF* family member *Aco006567* was expressed in the nucellar cells of MMC-stage ovules (Fig. 9A). Moreover, the ethylene response factor *Aco014268* was also expressed in the epidermis, middle layer, and tapetum of PMC-stage stamens (Fig. 10C).

In summary, coupling a detailed developmental characterization of germline development with comparative transcriptomics allowed the identification of several genes that likely play key roles during germline specification in pineapple. We observed that most of the known regulators of germline development also appear to play important roles in pineapple, suggesting that the gene regulatory networks controlling germline specification are well conserved among plant species. Our approach also identified new regulatory candidates. Ascertaining and understanding the molecular functions of these genes during pineapple germline specification will nevertheless require further analyses.

## Materials and methods

### Plant material and growth conditions

Pineapple plants (cultivar MD2) were grown in a greenhouse at 28 °C ± 2 °C. When the plants reached approximately the 30-leaf stage, flowering was induced using 800 mg/l ethylene and 1% urea (w/v). An induction solution (~20 ml/plant) was used for flower induction, and watering was stopped for a week after the treatment. The formation of flower buds was observed at approximately five weeks posttreatment.

### Cytological analysis

Fresh flower buds of different sizes were measured and then fixed in FAA (50% ethanol, 5% (v/v) acetic acid, 3.7% (v/v) formaldehyde) overnight. The ovules or anthers were placed in a drop of chloral hydrate solution (8:2:1, chloral hydrate: H_2_O: glycerol) on a slide and observed under DIC optics with a ZEISS Axio Imager A2^15^.

### Morphometric analysis of the floral bud

For flower morphology, buds with bracts were cut; the maximum length of the proximal-distal axis and the horizontal width were used as a reference for measurement. The full lengths from visible petals were measured. Different bud sizes corresponding to the ovule and pollen development stages are shown in Table 1.

### Chromosome spreading

Fixed anthers were first washed with fixative (3 ethanol:1 glacial acetic acid) and then with citrate buffer (pH 4.5) before being incubated in an enzyme digestion mixture. The enzyme digestion mixture contained 0.3% cellulase/pectinase/driselase. Stock solutions of cellulase (C-9422; Sigma–Aldrich), pectinase (P-4716; Sigma–Aldrich), and driselase (D-9515; Sigma–Aldrich) were prepared in a solution containing 10 mM citrate, pH 4.5, and 45% glycerol and stored at −20 °C. Tweezers were used to crush and transfer the siliques to the slides. Ten milliliters of 60% acetic acid was added to the slide before it was placed on a hot plate at 45 °C for 1 min while stirring with a needle. An additional 10 ml of 60% acetic acid was added to the slide, which was removed from the hotplate, before the addition of 200 ml of cold 3:1 fixative. The fixative was removed, and the slide was dried with a hair drier. Chromosome staining was performed using 1 mM 4′6-diamidino-2-phenylindole (DAPI) in PBS/50% glycerol. Chromosomes were observed on a ZEISS Axio Imager A2 using a 365-nm excitation, 420-nm long-pass emission filter.

### Whole-mount protein immunolocalization

The ovules of different meiosis stages were fixed for at least 3 h in 4% (w/v) paraformaldehyde (prepared in 1 × PBS and 2% Triton X-100) on ice. After fixation, the samples were washed three times with 1 × PBS, and the ovules were transferred onto a slide coated with poly-L-lysine. The ovules were quickly arranged in embedding solution, pressed carefully after covering with a clean coverslip, and kept for 20 min to allow polymerization at room temperature. After 20 min, the coverslip was removed gently, and the ovules were digested in an enzymatic solution (1% driselase, 1% pectolyase and 0.5% cellulose in 1 × PBS) at 37 °C for 60 min. Following three washes with 1 × PBS/0.2% Triton X-100, the ovules were incubated overnight at 4 °C with a DMC1 primary antibody (ABclonal Technology, Cat # AS044) at a dilution of 1:200. The primary antibody was removed by washing with 1 × PBS/0.2% Triton X-100 for at least 6 h (the solution was changed every hour). The ovules were then incubated overnight with a secondary antibody (Alexa Fluor 488) used at a dilution of 1:200 at 4 °C. The secondary antibody was washed off in the same way as the primary antibody, and the slides were counterstained with 50 μl of PI (500 μg/ml) for 20 min. After washing the slides 3 times with 1 × PBS/0.2% Triton X-100, the slides were mounted with one drop of a prolonged anti-fade solution^41^. The slides were then observed under a Leica SP8 confocal microscope by selecting the appropriate laser. For immunostaining, the experiments were repeated using three biological replicates.

### RNA-seq analysis

We obtained the raw RNA-seq data of Ovule_1, Ovule_2, Ovule_3, Ovule_4, Ovule_5, Ovule_6, and Ovule_7 and Stamen_1, Stamen_2, Stamen_3, Stamen_4, and Stamen_6 from the European Nucleotide Archive (ENA) under accession number PRJEB38680. The pineapple leaf, root and mature flower RNA-seq data were downloaded from https://de.iplantcollaborative.org/de/?type=data&folder=/iplant/home/cmwai/coge_data/Pineapple_tissue_RNAseq. Raw reads were filtered by removing the low-quality and adapter sequences by Trimmomatic software with several parameters (PE; LEADING:3; TRAILING:3; SLIDINGWINDOW:4:15 and MINLEN:36)^79^. Clean reads were aligned to the reference genome with TopHat^80^. We used the pineapple genome as the reference (https://genome.jgi.doe.gov/portal/pages/dynamicOrganismDownload.jsf?organism=Acomosus). To identify differentially expressed genes, we used two methods, Cuffdiff and DEseq2^80,81^. First, the alignment results were processed using Cufflinks for gene quantification, and Cuffdiff was then used to identify the differentially expressed genes (abs (log2 (fold change)) > = 2, q value < = 0.05) between each sample. Second, we used featureCounts^82^ to calculate the read counts for each gene and then used the DEseq2 program to detect the differentially expressed genes (abs (log2(fold change)) > = 2, p-adjusted < = 0.05). Finally, the common gene sets obtained by Cuffdiff and DEseq2 were retained for further analysis.

### Enrichment analysis

GO enrichment analysis of DEGs was performed using AgriGO (http://systemsbiology.cau.edu.cn/agriGOv2/index.php#)^83^ with a *P* value cutoff (10^−5^) to screen the significantly enriched GO terms. The AgriGO parameters were as follows: statistical test method, Fisher; Muti_test adjustment method, Yekutieli (FDR under dependency); significance level, 0.05. We downloaded pineapple GO annotations from the PGD database (http://pineapple.angiosperms.org/pineapple/html/index.html). We used BLASTP software to identify and define the homologs between pineapple and Arabidopsis. First, we identified the 5 homologs of *Arabidopsis* genes in pineapple in using BLASTP software with two parameters (Evalue < 1e-5 and max_target_seqs = 5). Then, we determined the most homologous genes by constructing a phylogenetic tree with these genes. Multiple sequence alignments were carried out using the Muscle^84^ program with default parameters. Phylogenetic analysis was performed with MEGA 6.06^85^ software via the neighbor-joining method. An example is shown in Supplementary Fig. S3. Kyoto Encyclopedia of Genes and Genomes (KEGG) assigns genes from many organisms to pathways. For KEGG pathway enrichment analysis, TBtools^86^ software was employed. All GO and KEGG analysis results were generated and shown by ggplot2 (a package of R software).

### qRT–PCR analysis

qRT–PCR analysis was performed using three biological replicates from independently grown plant materials. RNA from a pool of primary ovules or anthers was prepared using the RNeasy Plant Mini Kit (Qiagen), followed by cDNA synthesis with the First Strand cDNA Synthesis Kit (Promega). qRT–PCR was carried out in the presence of SYBR Green (Takara) using a Bio–Rad CFX96 Touch™ real-time PCR machine (Bio–Rad, Singapore). Relative expression was calculated with the 2^−△△CT^ method using the reference gene *Tubulin* (Aco023422). Statistical analysis was performed with Microsoft Excel software based on the results of three biological replicates, with the standard deviations being shown as error bars.

### In situ hybridization

For in situ hybridization, DNA fragments were used for RNA probe synthesis. The desired fragments were amplified and cloned into the pTA2 vector (Toyobo) as reported previously^15,63^. The primers used for probe amplification are listed in Supplementary Table S8. For pineapple MMC-stage ovules and PMC stage stamens, samples were fixed in RNase-free 4% (w/v) paraformaldehyde overnight (12–15 h) at 4 °C. The samples were dehydrated and cleared through a series of ethanol and chloroform gradients, respectively, and then embedded in Paraplast Plus (Sigma). All samples were sectioned at a thickness of 8–10 μm using an RM2245 rotary microtome (Leica). Hybridization experiments were performed as previously described^63,87^.

## Supplementary information

supplemental figures

supplemental table

## Data Availability

The datasets used and/or analyzed during this study are available from the corresponding author on reasonable request. RNA-seq raw data were deposited into the European Nucleotide Archive (ENA) under accession number PRJEB38680.
