# Supplementary material for: Characterization of germline development and identification of genes associated with germline specification in pineapple
Source: Hortic Res. 2021 Nov 1;8:239. doi: 10.1038/s41438-021-00669-x (PMC8558326; doi:10.1038/s41438-021-00669-x)

## Slide 1
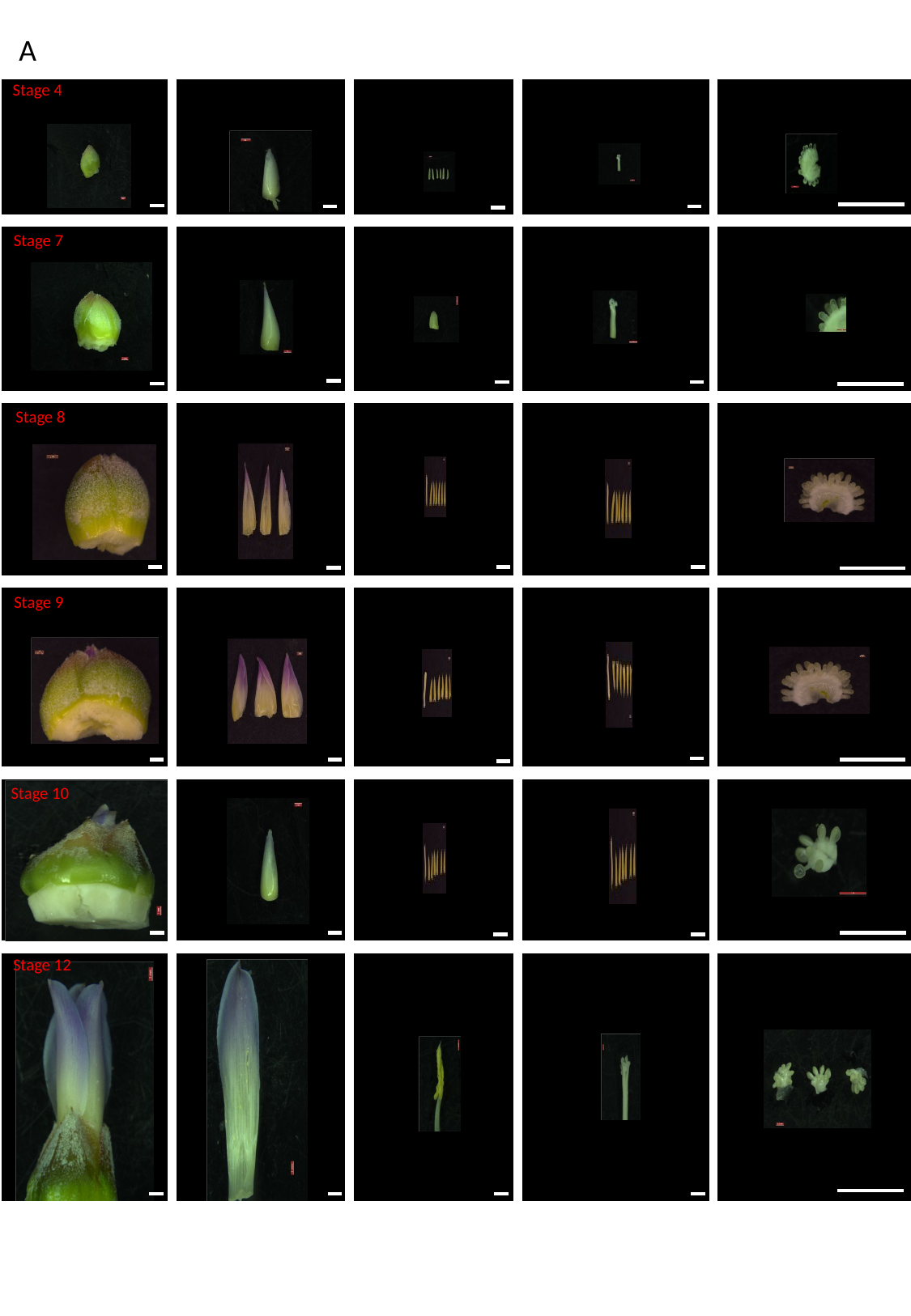

A
Stage 4
Stage 7
Stage 8
Stage 9
Stage 10
Stage 12

## Slide 2
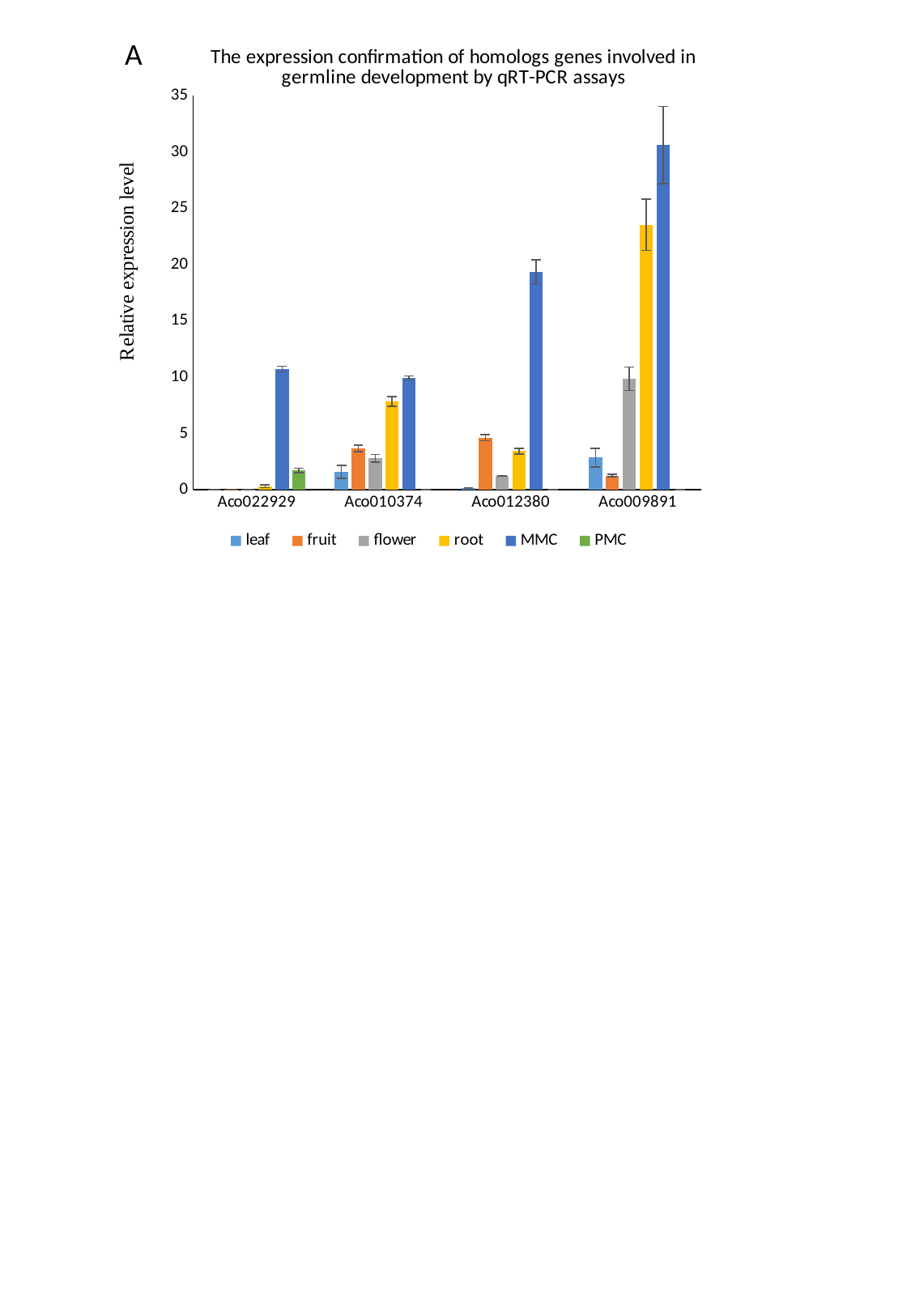

### Chart: The expression confirmation of homologs genes involved in germline development by qRT-PCR assays
| Category | leaf | fruit | flower | root | MMC | PMC |
|---|---|---|---|---|---|---|
| Aco022929 | 0.006136704619153942 | 0.019322291422670775 | 0.020045191434363924 | 0.32070275249479296 | 10.713957116853422 | 1.7124963183103628 |
| Aco010374 | 1.577530173977797 | 3.6688674499101257 | 2.7777714147170323 | 7.8217399323287395 | 9.92803978800079 | 0.0 |
| Aco012380 | 0.13340653074859948 | 4.626809451454176 | 1.211281239560427 | 3.423719963330471 | 19.361606878797833 | 0.0 |
| Aco009891 | 2.8502199883473787 | 1.2581327838653351 | 9.837702842694043 | 23.54014322316647 | 30.617842343794525 | 0.0 |A

## Slide 3
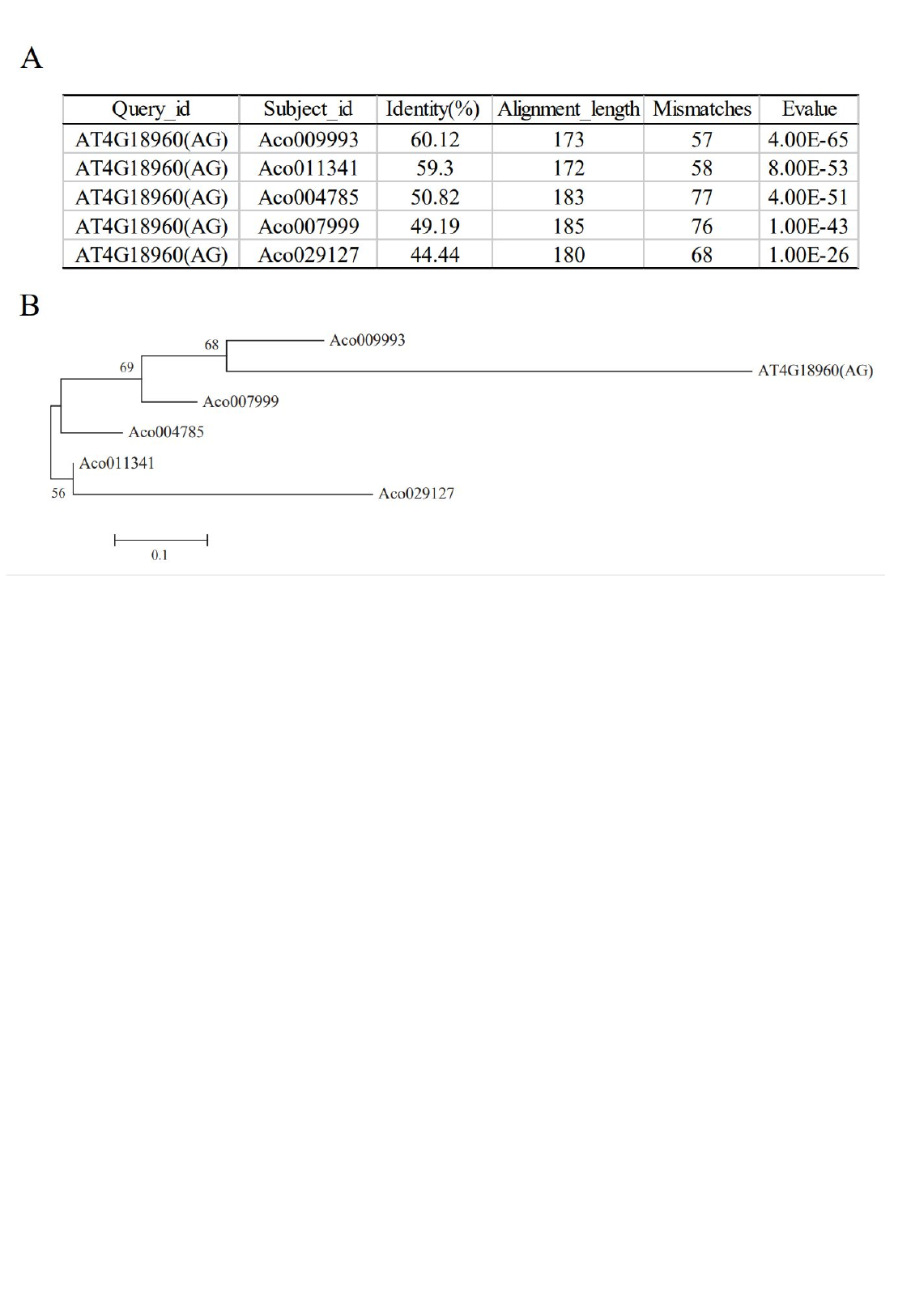

Supplement: Supplementary file 1 — supplemental figures [file 41438_2021_669_MOESM1_ESM.pptx]
